# Molecular cytogenetics of tragelaphine and alcelaphine interspecies hybrids: hybridization, introgression and speciation in some African antelope

**DOI:** 10.1098/rsbl.2015.0707

**Published:** 2015-11

**Authors:** T. J. Robinson, H. Cernohorska, E. Schulze, A. Duran-Puig

**Affiliations:** 1 Evolutionary Genomics Group, Department of Botany and Zoology, University of Stellenbosch, Stellenbosch, South Africa; 2 Veterinary Research Institute, Brno, Czech Republic; 3 Free State Department of Economic Development, Tourism and Environmental Affairs, Bloemfontein, South Africa

**Keywords:** cross-species chromosome painting, antelope hybrids, evolution

## Abstract

Hybridization can occur naturally among diverging lineages as part of the evolutionary process leading to complete reproductive isolation, or it can result from range shifts and habitat alteration through global warming and/or other anthropogenic influences. Here we report a molecular cytogenetic investigation of hybridization between taxonomically distinct species of the Alcelaphini (*Alcelaphus buselaphus* 2*n* = 40 × *Damaliscus lunatus* 2*n* = 36) and the Tragelaphini (*Tragelaphus strepsiceros 2n* = 31/32 × *Tragelaphus angasii* 2*n* = 55/56). Cross-species fluorescence *in situ* hybridization provides unequivocal evidence of the scale of karyotypic difference distinguishing parental species. The findings suggest that although hybrid meiosis of the former cross would necessitate the formation of a chain of seven, a ring of four and one trivalent, the progeny follow Haldane's rule showing F_1_ male sterility and female fertility. The tragelaphine F_1_ hybrid, a male, was similarly sterile and, given the 11 trivalents and chain of five anticipated in its meiosis, not unexpectedly so. We discuss these findings within the context of the broader evolutionary significance of hybridization in African antelope, and reflect on what these hold for our views of antelope species and their conservation.

## 1. Introduction

Interspecific hybridization, particularly within Bovidae (antelope, cattle, sheep and goats), is not an infrequent observation [1], highlighting the relatively shallow divergences of many of the terminal taxa in bovid phylogenetic trees [2]. It can result from changes in species distributions due to alteration of habitat as a consequence of global change and anthropogenic influence [3], and can variably affect the rate of differentiation and impact at different stages of divergence [4]. Additionally, in the spatially constrained settings almost invariably associated with modern conservation and game farming practices, hybridization among species poses different threats to previously distinct populations, often with unintended outcomes.

We recently had the opportunity to analyse the chromosomes of two purported interspecific hybrids. One, an *Alcelaphus buselaphus* (red hartebeest) × *Damaliscus lunatus* (tsessebe) F_1_ male that was culled on a wildlife reserve as part of an intervention conducted by a regional conservation agency in the Free State Province of South Africa. This was done to test historic [5] and anecdotal reports of the species hybridization (electronic supplementary material). The second, a *Tragelaphus strepsiceros* (greater kudu) × *Tragelaphus angasii* (nyala) F_1_ male arose from hybridization on a game farm in the North West Province of South Africa [6]. We report the outcome of a molecular cytogenetic investigation of these hybrid animals to raise awareness of the broader evolutionary significance of hybridization in African antelope, the variability in evolutionary time distinguishing hybridizing species pairs and the implications this holds for antelope diversity and conservation.

## 2. Material and methods

Tissues collected for the study were derived from adult male F­_1_ hybrids. The culture of fibroblasts, G-banding of chromosomes as well as the generation of painting probes for fluorescence *in situ* hybridization (FISH) followed established protocols. Cross-species FISH, using cattle (*Bos taurus;* BTA) whole chromosome painting probes, was conducted to confirm (and in some instances correct) chromosomal homology among parental taxa as well as their hybrids (electronic supplementary material). Chromosomes are numbered according to cattle standard [7].

## 3. Results

### (a) Cytogenetics of the *Alcelaphus buselaphus × Damaliscus lunatus* F_1_

**Figure 1.**
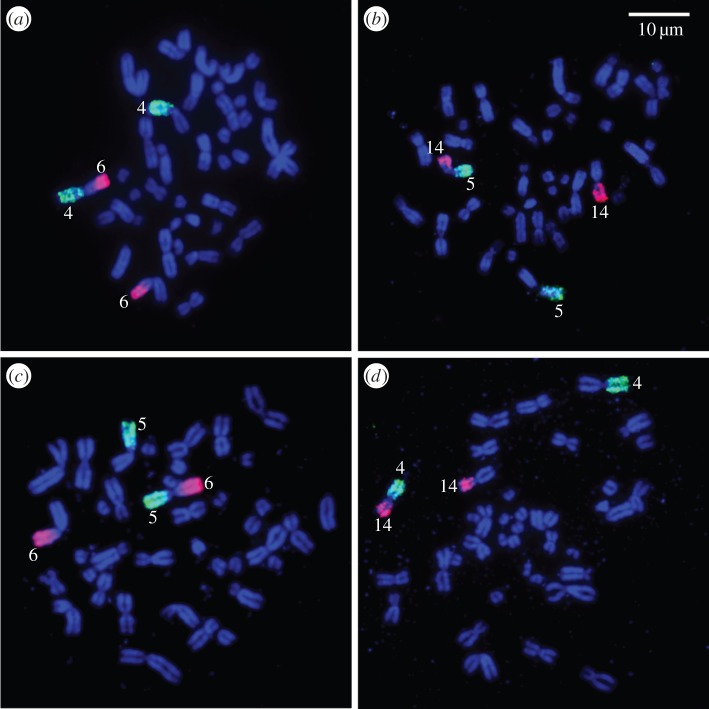
FISH using cattle (BTA) chromosome painting probes to validate some of the brachial combinations resulting from Robertsonian (Rb) fusions in the chromosomal complement of the F_1_ hybrid of a cross between a red hartebeest male and a female tsessebe. (*a*) Rb4;6, (*b*) Rb5;14, (*c*) Rb5;6 and (*d*) Rb4;14.

The G-banded karyotypes of both parental species have previously been published and are consequently not presented (electronic supplementary material, table S1). The red hartebeest (*A. buselaphus*) has 2*n* = 40 and the tsessebe (*D. lunatus*) 2*n* = 36. Meiosis in male red hartebeest would yield gametes with 20,Y and female tsessebe gametes with 18,X; the presumptive F_1_ hybrid with 2*n* = 38 is consistent with this (table 1; electronic supplementary material, figure S1) as is its phenotype which showed a relatively subtle mix of traits from both parental taxa. However, the differences between the two species' karyotypes are quite marked. There are six metacentric bivalents and five acrocentric bivalents in common but there are several monobrachial combinations (figure 1) that would result in complex multivalents in the hybrid's meiosis. These include a chain of seven (13–13.15–15.11–11.23–23.22–22.20–20), a ring of four (*14.5–5.6–6.4–4.14*) and one trivalent (18–18.24–24). Clinical and reproductive analyses indicate the hybrid to be sterile but, and in contrast to the male, several female tsessebe × red hartebeest F_1_ hybrids from the same herd were fertile (see the electronic supplementary material, figure S3)—an observation that would be consistent with Haldane's rule (i.e. ‘When in the F_1_ offspring of two different animal races one sex is absent, rare or sterile, that sex is the heterozygous sex’ [8]). Mechanisms responsible for sex-biased hybrid dysfunction (sterility and inviability) remain largely undefined. Among others they include the ‘large X effect’ (the X chromosome contributes disproportionately to sterility in hybrids relative to other chromosomes [9], the independent genetic control of meiosis and gametogenesis in the two sexes ([10], and a lack of checkpoint control in female meiosis that in males is more stringent [11].

**Table 1.** Chromosomal composition of alcelaphine and tragelaphine species pairs and their F1 hybrids, as identified by G-banding and confirmed by comparative FISH. Chromosomes are numbered according to the cattle standard [7].

parental species	chromosomal composition
**(haploid chromosome number)**	**F1 hybrids**
**tribe Alcelaphini**	**alcelaphine F1 hybrid (2*n*=38; see figure S1)**
*Alcelaphus buselaphus* (20,Y): 1;10, 2;25, 3;19, 4;6, 5;14, 7;9, 8;17, 11;15, 12;16, 22;23, 13, 18, 20, 21, 24, 26, 27, 28, 29, Y *Damaliscus lunatus* (18,X): 1;10, 2;25, 3;19, 4;14, 5;6, 7;9, 8;17, 11;23, 12;16, 13;15, 18;24, 20;22, 21, 26, 27, 28, 29, X	1;10, 1;10, 2;25, 2;25, 3;19, 3;19, 4;14, 4;6, 5;6, 5;14, 7;9, 7;9, 8;17, 8;17, 11;15, 11;23, 12;16, 12;16, 13;15, 13, 18;24, 18, 24, 20;22, 20, 21, 21, 22;23, 26, 26, 27, 27, 28, 28, 29, 29, X, Y
**tribe Tragelaphini**	**tragelaphine F1 hybrid (2*n*=43; see figure S2)**
*Tragelaphus strepsiceros* (15,t(Y;13)): 1;29, 3;10, 4;5, 6;20, 7;18, 8;17, 9;27, 11;23, 12;16, 14;26, 15;28, 19;21, 24;22;2, 25, Y;13 *Tragelaphus angasii* (28,X): 1, 3, 4; 5, 6, 7, 8, 9, 10, 12, 13, 14, 11;22;2, 15, 16, 17, 18, 19, 20, 21, 22, 23, 24, 25, 26, 27, 28, 29, X	11;22;2, 24;22;2, 24, 1;29, 1, 29, 3;10, 3, 10, 4;5, 4, 5, 6;20, 6, 20, 7;18, 7, 18, 8;17, 8, 17, 9;27, 9, 27, 11;23, 23, 12;16, 12, 16, 14;26, 14, 26, 15;28, 15, 28, 19;21, 19, 21, 25, 25, X, Y;13, 13

### (b) Cytogenetics of the *Tragelaphus strepsiceros × Tragelaphus angasii* F_1_

**Figure 2.**
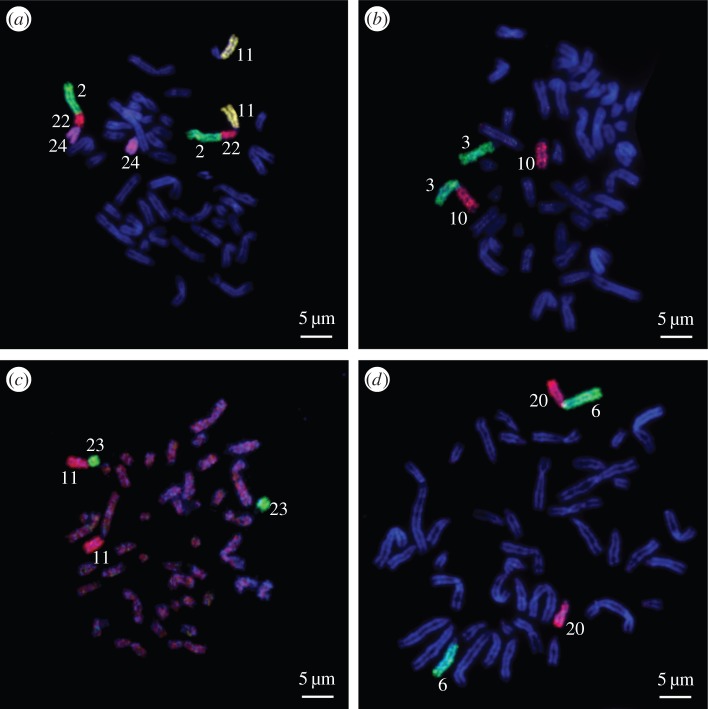
FISH using cattle (BTA) chromosome painting probes to validate some of the brachial combinations resulting from Robertsonian (Rb) fusions in the chromosomal complement of the F_1_ hybrid of a cross between a greater kudu male and a female nyala. (*a*) Rb2;22;11 and Rb2;22;24, (*b*) Rb3;10, (*c*) Rb11;23 and (*d*) Rb6;20.

Banded karyotypes have been published for both Tragelaphini parental species and are not repeated (electronic supplementary material, table S1). The greater kudu (*T. strepsiceros*) has 2*n* = 31/32 and meiosis in males would yield 15,t(Y;13) gametes and, in the female nyala (*T. angasii*) (2*n* = 56,XX), gametes with 28,X—both consistent with the 2*n* = 43 complement observed in the F_1_ hybrid male (table 1; electronic supplementary material, figure S2). FISH analysis of fusions characterizing the parental species provides unequivocal identification that chromosomal rearrangements in the parental species are present in the F_1_ hybrid (figure 2).

As with the alcelaphine hybrid, the greater kudu × nyala cross was considered sterile following detailed clinical and reproductive potential assessments [6]. This may be anticipated given the 11 trivalents (4–4.5–5; 3–3.10–10; 1–1.29–29; 6–6.20–20; 7–7.18–18; 8–8.17–17; 12–12.16–16; 9–9.27–27; 19–19.21–21; 14–14.26–26; 15–15.28–28) and chain of five (24–24.22.2–2.22.11–11.23–23) expected in the hybrid's meiosis. The two parental species have a single autosomal bivalent in common (25–25; table 1).

## 4. Discussion

Although both instances of hybridization reflect anthropogenic influence (controlled access to conspecifics in the red hartebeest × tsessebe cross, low densities of species pairs in the case of the greater kudu × nyala cross; details in the electronic supplementary material), it is not known how frequently these occur in nature and whether the incidence has been heightened through range extension (due to translocation, principally through game farming and/or habitat modification). It is noteworthy, however, that the historic ranges of the red hartebeest and tsessebe overlapped naturally north of South Africa, and in areas to the north of the Orange River in the northwestern parts of the Free State Province, South Africa [12,13]. The greater kudu and nyala show extensive sympatry in the southeastern parts of the southern African subregion [14]. Given these distribution patterns, it seems that although hybridization can occur between these taxa, the genetic integrity of the species pairs (through introgression of genetic and phenotypic characters from one species into the other) is not significantly compromised, even if female-mediated gene flow seems possible in the case of red hartebeest × tsessebe (electronic supplementary material, figure S3). Distinctiveness is likely underpinned by assortative mating and habitat specificity (*D. lunatus* prefers fringes of grassland that merge into woodlands, while *A. buselaphus* occurs in open areas and avoids closed woodland [14]). Interestingly, there is one recorded instance of a red hartebeest × tsessebe hybrid observed in a natural setting [5] that predates anthropogenic influence, suggesting that hybridization is insufficient in nature to homogenize the nuclear gene pool, and that progeny of mixed parent pairs probably have reduced fitness under competitive conditions.

In the broader sense, however, these data serve to highlight several important considerations associated with the radiation of many of the African antelope. Using a recently published multi-calibrated molecular phylogeny to broadly affix divergence times of hybridizing species pairs, it would appear that the viable F_1_ offspring detected here result from crosses between species that last shared common ancestry in fairly deep evolutionary time—6.1–7.3 Ma for *T. strepsiceros* and *T. angasii* and 3.2–5.1 Ma for *Damaliscus* (proxy for *D. lunatus*) and *A. buselaphus* (see fig. 1 in [2] for 95% range for node ages). There are other documented instances of hybridization in both tribes. In the Tragelaphini: *Tragelaphus scriptus* × *Tragelaphus* (*Taurotragus*) *oryx* (4.5–5.4 Ma; 6.1–7.3 Ma), *Tragelaphus spekei* × *Tragelaphus* (*Boocerus*) *euryceros* (3.0–3.6 Ma) and *T. spekei* × *Tragelaphus imberbis* (6.8–8.0 Ma). Hybrid offspring were recorded from all three crosses (a fertile female hybrid in the case of *T. spekei* × *T. euryceros*; [1]). In the Alcelaphini, hybrids have been recorded between *Connochaetes taurinus* × *Connochaetes gnou* ([15]; 0.7–1.6 Ma) and *A. buselaphus* × *Pygargus phillipsi phillipsi* ([16]; 3.2–5.1 Ma). F_1_ hybrids are fertile in the former and sterile in the latter cross.

In the relatively recent past, there has been a move away from defining species on grounds of reproductive isolation (the biological species concept or BSC) to new species definitions (such as the phylogenetic species concept) that view species as a continuum of separately evolving lineages that extend from ecological races to hybridizing species and, ultimately, to species that no longer cross [17,18] (see [19] for discussion in ungulates). In other words, there is a loss of the tendency to hybridize that is almost clock-like and, as a consequence, much of the evolution leading to reproductive isolation occurs while gene flow persists [20]. In fact, there is considerable evidence to suggest that hybridization, which is individually rare, is relatively frequent in plants (25%) but less so in animal species (10%). Six per cent of European mammal species are thought to hybridize [20]. It is important therefore to understand the context in which hybridization occurs and, where possible (and/or necessary), manage the consequences appropriately.

We anticipate that hybridization, possibly with attendant hybrid zones [21], may be detected in some antelope. This would be in keeping with hybridization being a natural phenomenon in their evolutionary history, a process that is thought generally to have led to the persistence of species through periods of climate change [3]. African antelope likely to exhibit hybridization are those where population structure depends on geographical distance moulded by fragmented habitats (particularly forests, grassland patches and wetlands that are subjected to repeated interglacial cycles of expansion and contraction [22–24]). These include Reduncini (waterbuck and allies, particularly within *Redunca* 3.4–5.2 Ma and *Kobus* 2.4–3.4 Ma), plains antelope such as the Antilopini (gazelles, particularly in *Gazella*: 2.3–3.1 Ma), forest taxa such as the Cephalophini (duikers, particularly in *Cephalophus*: 5.6–7.2 Ma) and among Hippotragini (4.6–6.7 Ma [25], 1.6–2.4 Ma [1]). There are recorded [1,15,16,25] and recent instances of hybridization (electronic supplementary material, figure S4) among species of all the tribes indicated above. Although this is clearly not likely to lead to introgression in all instances, these data are, nonetheless, of particular *ex situ* conservation concern. In more natural settings (including game farms), where the morphological distinctiveness of hybrids is relatively subtle (see for example the electronic supplementary material, figure S3), detection is probably underestimated, confounding detailed scrutiny and appropriate interventions, should these be required.

In general, although hybridization is more likely among closely related species/taxa (possibly leading to adaptive introgression, [4]), there is considerable temporal variation in the likelihood that should hybridization be successful, reproductive impairment will result. These considerations add to the complexity of formulating policy to manage biota in changing landscapes, particularly where increased contact between ecologically segregated species may result, or historic distributions and species habits are manipulated or ignored. They are also a challenge to agencies whose conservation policies are largely founded on the biological species concept (reproductive isolation).

## Supplementary Material

Electronic supplementary material Robinson et al

## Supplementary Material

15 20 Oct Robinson et al Referee responses and rebuttal.docx

## Data accessibility

Electronic supplementary information supporting this manuscript has been uploaded.
