# Supplementary material for: Molecular cytogenetics of tragelaphine and alcelaphine interspecies hybrids: hybridization, introgression and speciation in some African antelope
Source: Biol Lett. 2015 Nov;11(11):20150707. doi: 10.1098/rsbl.2015.0707 (PMC4685540; doi:10.1098/rsbl.2015.0707)

Supplementary information - TJ Robinson, H Cernohorska, E Schulze, A Duran-Puig

Provenance, Cell culture, G-band and FISH analysis:

Provenance: Following reports of interspecific hybridization of Red hartebeest (*Alcelaphus buselaphus*) and Tsessebe *(Damaliscus lunatus*) at the Itala Game Reserve (27 ° 30 m' 14" S   31 ° 18' 31" E) in KwaZulu-Natal, South Africa (Rushworth *in litt.*), and the historic report of a similar situation that predated anthropogenic influence [1], a conservation intervention that included controlled access to conspecific mates was initiated in the Free State Province where both taxa are important game species. This led to the establishment of F1 Red Hartebeest x Tsessebe hybrids (1 male and 4 females) at the Rustfontein Dam Nature Reserve (29° 16' 44" S   26° 36' 00" E) in 2007. Once adult, the F1 male hybrid had exclusive access to a herd comprising hybrid and Tsessebe females for consecutive breading seasons without producing offspring (substantiating the reproductive and clinical evidence of infertility, see below). Following its replacement by a Red Hartebeest bull in January 2013, the female F1 hybrids and two Tsessebe produced calves (Supplementary Fig S3). See [2] for information on the Greater kudu x Nyala hybrid.

Cell culture: Fibroblast cultures were established from skin and callus biopsies of adult male hybrids taken in the field: (i) a Red hartebeest x Tsessebe hybrid (resulting from a male *A. buselaphus* x female *D. lunatus* cross) collected at the Rustfontein Dam Nature Reserve (above), and (ii) a Greater kudu x Nyala hybrid (from a male *T. strepsiceros* x female *T. angasii* cross) collected from a private game farm in the North West Province of South Africa (October 2013). Biopsy material was grown at 37 °C in Dulbecos Modified Medium (DMEM) enriched with 15% bovine fetal serum. Cell harvest followed conventional procedures; chromosome preparations were aged at 65 °C overnight before G-banding with trypsin. Karyotypes derived from cryopreserved fibroblast cells of the two parental species were similarly prepared and used to facilitate analysis of the hybrids' chromosomes. Chromosomal nomenclature follows that for cattle [3].

Fluorescence in situ *hybridization (FISH)*: Cross-species FISH was used to confirm (and in some instances correct) chromosomal homology among parental taxa and hybrids. Cattle whole chromosome painting probes were produced by microdissection (PALM Microlaser system, Carl Zeiss MicroImaging GmbH, Munich, Germany). Laser dissected DNA was amplified by degenerate oligonucleotide primed polymerase chain reaction (DOP-PCR) and labeled during the secondary PCR with Orange–dUTP or Green-dUTP (Abbott, Ill, USA) [4].

1. Selous FC. 1893 On a hybrid antelope. *Proc Zool Soc* **1**, 1-2.

2. Dalton DL, Tordiffe A, Luther I, Duran A, van Wyk AM, Brettschneider H, Oosthuizen A, Modiba C, Kotze A. 2014 Interspecific hybridization between greater kudu and nyala. *Genetica* **142**, 265-271.

3. ISCNDB 2000. 2001 International system for chromosome nomenclature of domestic bovids. *Cytogenet Cell Genet* **92**, 283–299.

4. Kubickova S, Cernohorska H, Musilova P, Rubes J. 2002 The use of laser microdissection for the preparation of chromosome specific painting probes in farm animals. *Chromosome Res* **10**, 571–577.

Supplementary Table S1: Chromosomal data and source references for the hybridizing parental species.

Species	Diploid number	Reference
*Damaliscus lunatus**	2n = 36	[1], [2], [3], [4].
Alcelaphus buselaphus	2n=40	[5], [6], [3].
*T. strepsiceros***	2n=31/32	[1], [3], [7].
*T. angasii****	2n=55/56	[1], [3], [7].

*Our painting data on *D. lunatus* confirm those of [4] which show the second largest fusion chromosome as Rb2;25 and not Rb2;29 as in the earlier reports for this species [1], [2], [3].
** The Greater kudu (*T. strepsiceros*) has 2n=31/32.
*** The Nyala (*T. angasii*) has 2n=55/56.
The difference in 2n between sexes is due to a Y;13 translocation which results in both *T. strepsiceros* 31,X,t(Y;13) and *T. angasii* males 55,X,t(Y;13) invariably having one chromosome less than females.

In addition to the Y;13 fusion, which is characteristic of all Tragelaphini, the Greater kudu and Nyala karyotypes are characterized by a complex compound chromosomes thought to have arisen from a centric fusion of the orthologous cattle equivalents BTA 22 and BTA 2 followed by a tandem fusion with BTA 11 in the Nyala, and BTA 24 in the Greater kudu [7].

1. Gallagher DS Jr, Womack JE. 1992 Chromosome conservation in the Bovidae. *J Hered* **83**, 287–298.

2. Kumamoto AT, Charter SJ, Houck ML, Frahm M. 1996 Chromosome of *Damaliscus* (Artiodactyla, Bovidae): simple and complex centric fusion rearrangements. *Chromosome Res* **4**, 614–621.

3. O'Brien SJ, Menninger JC, Nash WG. 2006 *Atlas of Mammalian Chromosomes*. John Wiley and Sons, Hoboken.

4. Huang L, Jing M, Nie W, Robinson TJ, Yang F. 2011 Chromosome Homologies between Tsessebe (*Damaliscus lunatus*) and Chinese muntjac (*Muntiacus reevesi*)

Facilitate Tracing the Evolutionary History of *Damaliscus* (Bovidae, Antilopinae, Alcelaphini). *Cytogenet Genome Res* **132**, 264–270.

5. Buckland RA, Evans HJ. 1978 Cytogenetic aspects of phylogeny in the Bovidae. I. G-banding. *Cytogenet Cell Genet* **78**, 42–63.

6. Robinson TJ, Morris DJ, Fairall N. 1991 Interspecific hybridization in the Bovidae: sterility of *Alcelaphus buselaphus* x *Damaliscus dorcas* F1 progeny. Biol *Conserv* **58**, 345–356.

7. Rubes J, Kubickova S, Cernohorska H, Di Berardino D, Vahala J, Robinson TJ. 2008 Phylogenomic study of the Tragelaphinae (Family Bovidae) by cross-species chromosome painting with cattle paints. *Chromosome Res* **16**, 935–947.

Supplementary Fig. S1: G-banded karyotype of an F1 male hybrid (2n=38) resulting from a cross between a Red hartebeest male (*A. buselaphus*; 2n=40) and a female Tsessebe (*D. lunatus*; 2n=36). Chromosomes are numbered according to the cattle standard [1].

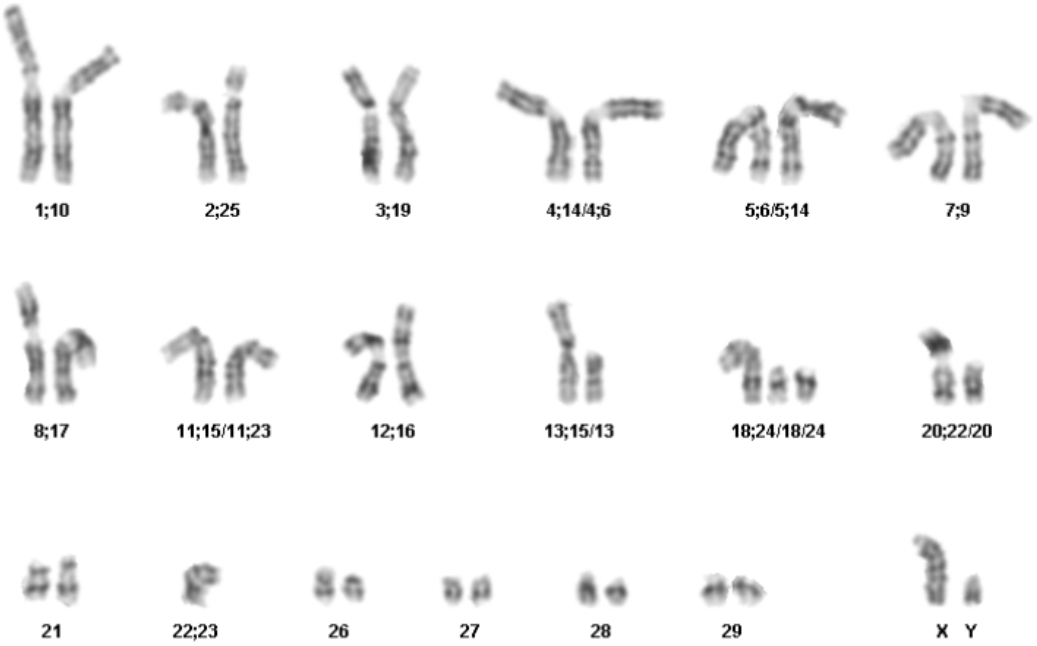

1. ISCNDB 2000. 2001 International system for chromosome nomenclature of domestic bovids. *Cytogenet Cell Genet* **92**, 283–299.

Supplementary Fig. S2: G-banded karyotype of an F1 male hybrid (2n=43) resulting from a cross between a Greater kudu male (*T. strepsiceros*; 2n=31) and a female Nyala (*T. angasii*; 2n=56). Chromosomes are numbered according to the cattle standard [1].

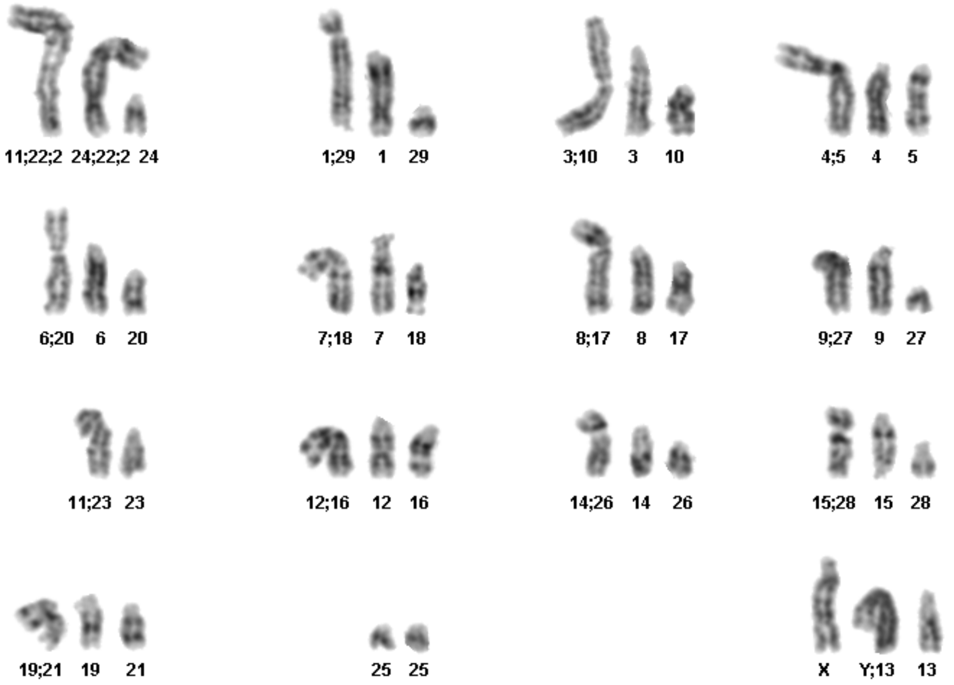

1. ISCNDB 2000. 2001 International system for chromosome nomenclature of domestic bovids. *Cytogenet Cell Genet* **92**, 283–299.

Clinical and reproductive analysis of the male hybrids:

(i) *T. strepsiceros x T. angasii*: see [1] for details.

(ii) *A. buselaphus* x *D. lunatus* F1: Several histological and clinical parameters were evaluated. (i) Fresh epididymal sperm smears were prepared in the field and examined microscopically for the presence of spermatozoa following Giemsa staining; (ii) testicular tissue was preserved in Bouin's fluid and subsequently embedded in paraffin wax and sectioned using a microtome. Photomicrographs were taken from mounted preparations stained with Ehrlich's haemotoxylin and eosin (below).

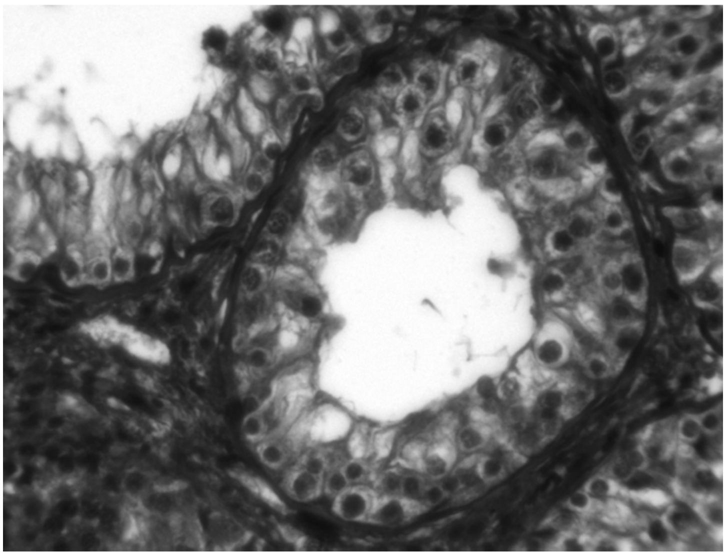

A paucity of germ cells and the absence of spermatids and spermatozoa in tubule cross-sections was noted. Although primary spermatocytes are evident in the preparation, meiotic activity was extremely limited. Additionally, the testis, epididymis, seminal vesicles and prostate were examined by pathologists who concluded that the male reproductive tract appeared normal, but there was a virtual absence of spermatogenesis (degeneration of some microtubules was also observed); the very few spermatozoa seen were considered abnormal.

1. Dalton DL, Tordiffe A, Luther I, Duran A, van Wyk AM, Brettschneider H, Oosthuizen A, Modiba C, Kotze A. 2014 Interspecific hybridization between greater kudu and nyala. *Genetica* **142**, 265-271.

Supplementary Fig S3: (a) Red hartebeest (*Alcelaphus buselaphus*) (photo credit R.J. van Aarde) and **(b)** Tsessebe (*Damaliscus lunatus*) showing the marked phenotypic differences between species. **(c)** A fertile F1 hybrid cow with calf (right); left front shows a hybrid cow calving (note protrusion under the tail). Both result from back-crossing to a Red hartebeest bull (photo credits **b** and **c** E. Schulze). Phenotypic identification of F1 hybrids (male and female) is based on subtle differences between the parental species. Hybrids are more similar to Tsessebe than to Red hartebeest. F1 hybrids of both sexes can be distinguished from Red hartebeest and Tsessebe through differences in horn shape and length. Hybrid colouring (body and face) is very similar to Tsessebe and is not a reliable diagnostic feature. Body size falls between that of Tsessebe and Red hartebeest making this less diagnostic than is horn shape and length.

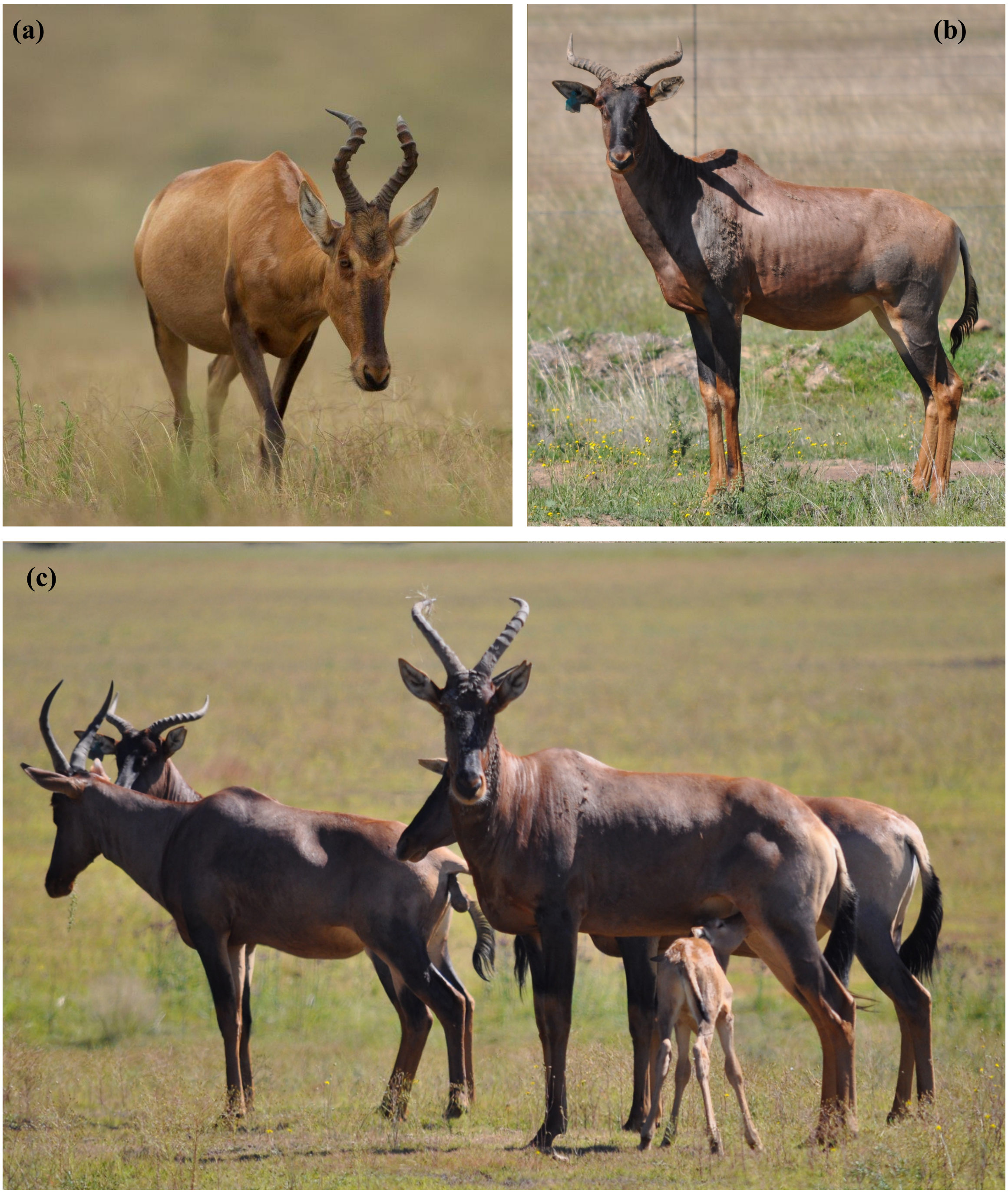

Supplementary Fig S4: A naturally occurring hybrid (right) from a *Kobus leche* (Red lechwe) x *K. ellipsiprymnus* (Waterbuck) cross approaching a female waterbuck in the Selinda Area of northern Botswana (photo credit R. Gärtner).

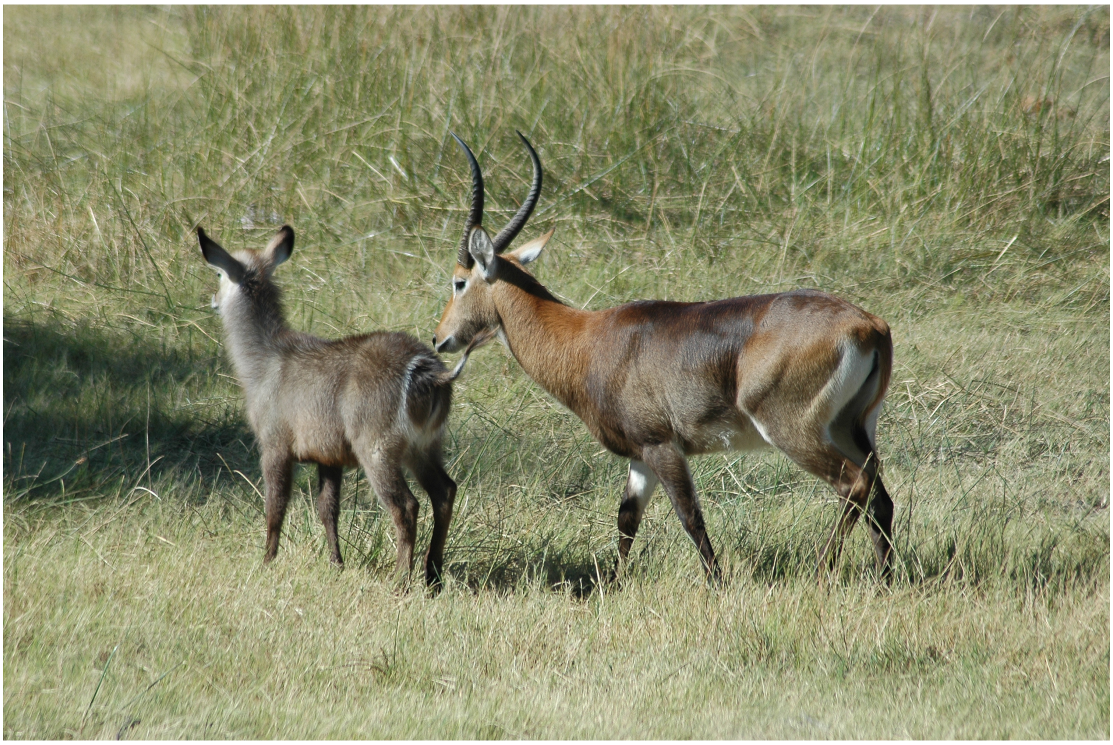

Supplement: Electronic supplementary material Robinson et al [file rsbl20150707supp1.pdf]
