# Supplementary material for: Molecular cytogenetics of tragelaphine and alcelaphine interspecies hybrids: hybridization, introgression and speciation in some African antelope
Source: Biol Lett. 2015 Nov;11(11):20150707. doi: 10.1098/rsbl.2015.0707 (PMC4685540; doi:10.1098/rsbl.2015.0707)
Supplement: 15 20 Oct Robinson et al Referee responses and rebuttal.docx [file rsbl20150707supp2.docx]

**Referee: 1 (received 15 October)**

Comments to the Author(s)

I think the paper is now in a position to be published in Biology Letters. The data presented and the discussion are interesting and the article is suitable for a wide audience.

**Referee: 2**

Comments to the Author(s)

From my point of view, it's absolutely essential to explain why the female on Supplementary Fig. S3 is hybrid. This is unclear for general readers as well for any zoologist non-specialist in antelope systematics. Maybe it would make sense to add two photos of the parent species and compare them with the hybrid.

We have included two additional images in the Electronic Supplementary Material that show the phenotypes of the hybridizing species. The F1 phenotype is predominantly that of the Tsessebe. The distinction of F1 hybrids is based on subtle differences that will probably not be evident to the non-specialist. Please see Supplementary Figures S3 a-c. We have included a statement on hybrid characteristics in the caption:

**Supplementary Fig S3:** **(a)** Red hartebeest (*Alcelaphus buselaphus*) (photo credit R.J. van Aarde) and **(b)** Tsessebe (*Damaliscus lunatus*) showing the marked phenotypic differences between species. **(c)** A fertile F1 hybrid cow with calf (right); left front shows a hybrid cow calving (note protrusion under the tail). Both result from back-crossing to a Red hartebeest bull (photo credits **b** and **c** E. Schulze). Phenotypic identification of F1 hybrids (male and female) is based on subtle differences between the parental species. Hybrids are more similar to Tsessebe than to Red hartebeest. F1 hybrids of both sexes can be distinguished from Red hartebeest and Tsessebe through differences in horn shape and length. Hybrid colouring (body and face) is very similar to Tsessebe and is not a reliable diagnostic feature. Body size falls between that of Tsessebe and Red hartebeest making this less diagnostic than is horn shape and length.

**Editorial Office (received Tuesday 20 October):**

Please use Vancouver style referencing in your supplementary file, and please ensure that your author contributions section meets all four criteria given here <https://royalsociety.org/journals/ethics-policies/openness/>.

We have revised the authors’ contributions to meet the journal’s authorship criteria (point 4) on accountability (lines 203-205).  The sentence reads: “All authors participated in data analysis, contributed to and approved the content of the final manuscript and acknowledge joint accountability for its content”.  This increased the word count to 2,929.

We have also amended the referencing used in the Electronic Supplementary Material to conform to the Vancouver style.
